# Traditional Chinese medicine as an adjunctive therapy for mild and common COVID-19: A systematic review and network meta-analysis

**DOI:** 10.1097/MD.0000000000027372

**Published:** 2021-10-08

**Authors:** Xiaozheng Wu, Wen Li, Zhong Qin, Lei Xue, Gao Huang, Zhenliang Luo, Yunzhi Chen

**Affiliations:** College of Preclinical Medicine, Guizhou University of Traditional Chinese Medicine, Guiyang, China.

**Keywords:** COVID-19, network meta-analysis, novel coronavirus pneumonia, systematic review, traditional Chinese medicine

## Abstract

**Background:**

Coronavirus disease 2019 (COVID-19) in many countries is still very serious. At present, there is no specific and effective drug for this disease. Traditional Chinese medicine (TCM) has played a great role in fighting against COVID-19. However, their effectiveness and safety are still obscure and deserve further investigation. The aim of the study was to evaluate the efficacy and safety of TCM assisted in conventional treatment in the treatment of mild and common COVID-19.

**Methods:**

PubMed, EMbase, MEDLINE, China National Knowledge Infrastructure Database, WANFANG DATA, and VIP Chinese Science and Technology Periodical Database were searched for randomized controlled trials (RCTs) and non-randomized controlled trials of TCM assisted in conventional treatment. The RCT research quality was evaluated by Cochrane 5.1.0 bias risk scale and the non-randomized controlled trial research quality was evaluated by Newcastle Ottawa scale, and the statistical analysis was conducted by Revman 5.3 and R software. The bias and sensitivity of the statistical results were analyzed by STATA 14.0. Registration number: CRD42020210619.

**Results:**

Fifteen studies were included with 7 RCT studies and 8 retrospective cohort studies, involving a total of 1623 patients. Compared with the control group, TCM can improve the main index clinical effective rate (odds ratio [OR] = 2.64, 95% Confidence interval (CI) [1.94,3.59], *P* < .00001). The results of Begg test (Pr > z = 0.266) and sensitivity analysis showed that the results were relatively stable. Toujie Quwen (OR = 4.9, 95%CI [1.9,14.0]), Shufeng Jiedu (OR = 2.9, 95%CI [1.5,5.7]), and Lianhua Qingwen (OR = 2.4, 95%CI [1.6,3.6]) were with the best. It can also improve the main clinical symptoms (fever, cough, fatigue, and the regression time of the 3 symptoms), severe conversion rate, and computed tomography improvement rate. Its safety was not significantly compared with conventional treatment. However, in terms of safety of a single TCM, Shufeng Jiedu (OR = –0.86, 95%CI [–1.89,0.09]) and Lianhua Qingwen (OR = –0.49, 95%CI[–0.94,–0.05]) were lower than those of conventional treatment.

**Conclusion:**

TCM as an adjuvant therapy combined with conventional treatment has good curative effect on mild and common type of COVID-19 patients. Its advantages lie in clinical efficacy and improvement of symptom group, and can prevent patients from transforming to severe disease. In terms of clinical efficacy and safety, Shufeng Jiedu and Lianhua Qingwen have obvious advantages, which are worthy of clinical promotion.

## 1 Introduction

A number of patients with unexplained pneumonia have been found in Wuhan, Hubei Province, China in December 2019. And later it was confirmed to be the result of a new coronavirus infection. On February 11, 2020, the International Committee on Classification of Viruses named the virus as severe acute respiratory syndrome coronavirus 2. Meanwhile, the World Health Organization named disease caused by it as the coronavirus disease 2019 (COVID-19).^[1]^ The epidemic situation in China has been basically controlled until today, but the situation in many foreign countries is still very serious. Novel coronavirus pneumonia is characterized by fever, cough, fatigue, expectoration, chest distress, dyspnea, muscle soreness, diarrhea, and so on. It can be classified as “epidemic disease” and “plague” in Chinese medicine.^[2–3]^ At present, there is no specific and effective drug for this disease, and the main clinical treatment is the symptomatic treatment. In China's experience in fighting against COVID-19, traditional Chinese medicine (TCM) has played a great role.^[4]^ In the process of novel coronavirus pneumonia, the therapeutic effect of Chinese medicine assisting western medicine has attracted wide attention, and related research reports are emerging. A large number of epidemiological studies have shown that the proportion of mild and common type of the disease is the largest.^[5]^ In China, “medical observation period” in the Novel Coronavirus Pneumonia Diagnosis and Treatment Plan (trial version 7th Edition)^[6]^ also recommended the Chinese medicine “Huoxiang Zhengqi capsule” (pill, water, and oral liquid), Jinhua Qinggan granule, Lianhua Qingwen capsule, Shufeng Jiedu capsule, and so on. However, TCM treatment has the characteristics of “individualization”, so it is difficult to formulate standard treatment details, which makes the evidence quality of clinical efficacy of TCM relatively weak. Therefore, it is necessary to carry out rigorous and objective quality evaluation for different types of clinical research, and the effectiveness analysis results obtained on this basis would be more convincing. Based on the published clinical research literature in China and abroad, this study conducted a systematic evaluation and network meta-analysis to understand the efficacy of TCM assisted western medicine in the treatment of COVID-19 more intuitively and evaluate the quality of evidence in current clinical trials of TCM to find potential problems and provide reference for the design and follow-up work of clinical trials of TCM.

## 2 Methods

This study has been registered in PROSPERO (https://www.crd.york.ac.uk/prospero/), registration number: CRD42020210619. The procedure of this protocol is based on PRISMA-P guidance.^[24]^

### 2.1 Inclusion criteria

Types of studies: randomized controlled trial (RCT) and non-randomized controlled trial (NRIs) of Chinese medicine treatment of COVID-19, whether blind method and allocation concealment were used or not, the language was Chinese or English. Types of participants: patients with mild and common type of COVID-19 who met the diagnostic criteria of COVID-19 diagnosis and treatment plan issued by the general office of the Chinese Health Commission and the office of the Chinese Medicine Bureau. Their gender, age, race, and nationality were not limited, and other serious diseases were excluded. Types of intervention: the treatment group: on the basis of the control group, TCM treatment was given. The dosage, dosage form, administration route, and method of Chinese medicine were not limited; the control group: conventional treatment, including oxygen therapy, antibiotics, antiviral, nutritional support treatment and other measures, drug treatment measurement and treatment were not limited. There is no time limit for intervention. Types of outcome measures: primary outcomes: clinical effective rate^[6]^; secondary outcomes: improvement rate and disappearance time of main symptoms; lung imaging (computed tomography [CT]) improvement rate; severe conversion rate; negative conversion rate of viral nucleic acid; incidence of adverse reactions.

### 2.2 Exclusion criteria

COVID-19 of heavy and dangerous heavy type; Self-control trial and non-control group study; Single use of conventional treatment or TCM; Summaries, case reports, experimental researches, and expert experiences; Only the one with the largest sample size and the most complete information was retained when the same study has been published for many times.

### 2.3 Database and search strategy

We searched PubMed, EMbase, MEDLINE, China National Knowledge Infrastructure Database and its COVID-19 special research results network launch platform, WANFANG DATA, and VIP Chinese Science and Technology Periodical Database. Chinese retrieval was based on subject words, keywords, literature tracing, and manual retrieval. First, “novel coronavirus pneumonia”, “2019-nCoV”, “COVID-19”, and “SARS-CoV-2” were used as the key words to search in WANFANG DATA. Then theme word “integrated Chinese and Western medicine”, “traditional Chinese medicine treatment”, “ traditional Chinese medicine”, and “prescription” were searched. The 2 searches were merged with “AND”. China National Knowledge Infrastructure Database has set up a network platform for the research results of COVID-19, thus it can be searched manually after selecting the “treatment” section in the platform. The English key words include“2019-nCoV” or “COVID-19” or “2019-nCoV pneumonia”, and “Drugs, Chinese Herbal” or “traditional Chinese herbal medicine” or “Chinese herb”. The search strategy of PubMed is presented in Table 1. The retrieval date is from the establishment of the database to July 31, 2020. At the same time, Baidu academic and Google Scholar search engines were used to search the relevant literature on the Internet.

**Table 1:** Example of PubMed search strategy.

Number	Search terms
#1	Mesh descriptor: (novel coronavirus) explode all trees
#2	((((((COVID-2019 [Title/Abstract]) OR 2019-nCoV [Title/Abstract]) OR novel coronavirus pneumonia [Title/Abstract]) OR COVID-2019 pneumonia [Title/Abstract]) OR 2019-nCoV pneumonia [Title/Abstract])
#3	Or 1-2
#4	Mesh descriptor: (traditional Chinese medicine) explode all trees
#5	((((((Drugs, Chinese Herbal [Title/Abstract]) OR traditional Chinese herbal medicine [Title/Abstract]) OR Chinese herb [Title/Abstract])
#6	Or 4–5
#7	3 and 6

As the epidemic situation continues, there are still clinical studies in progress. Before the evaluation results of this system are published, all databases will be retrieved again. If there are newly published studies, they will be directly included in the comprehensive treatment together with the previous studies.

### 2.4 Data collection and extraction

Two reviewers (Xiaozheng Wu and Wen Li) independently screened the literatures according to the inclusion and exclusion criteria. They furtherly read the full text of the studies that might meet the inclusion criteria to determine whether they could be really included after excluding the studies that obviously did not meet the inclusion criteria and then they cross checked them. In case of disagreement, they were discussed and resolved or handed over to the third party (Yunzhi Chen) for adjudication. If the report was not available or there was a lack of information, they would try to contact the author of the original text by email to obtain further relevant data. The design of data extraction table follows the principle of “PICOST” (participants, interventions, comparisons, outcomes, study design, time).

Data extraction contents include: general information: research ID, author, title, publication status, report sources, and fund support; methodology information: design, number of arms, random sequence generation, allocation concealment, blinding, incomplete outcome data, selective reporting, sample size calculation, and baseline comparability; participant information: diagnostic criteria, inclusion criteria, exclusion criteria, setting, population, sample size, age, gender, and course of disease; intervention information: name of intervention and comparation, syndrome differentiation of TCM, types of Chinese herbal medicines, dosage form, comparison, duration of treatment, and patient follow-up; outcomes; and adverse events.

### 2.5 Assessment of methodologic quality

#### 2.5.1 Quality evaluation of original research

The modified Jadad scale^[7]^ was used as the quality evaluation standard to evaluate the included RCT literature.

#### 2.5.2 Risk assessment of bias in the original study

The bias risk assessment tool recommended by Cochrane 5.1.0 was used to evaluate the included RCT studies, the contents are: generation of random assignment scheme; hidden grouping; blind method for patients and doctors; blind method for result evaluation; incomplete result data; selective result report; and other biases. The quality of the included studies was evaluated one by one. The quality of methodology was evaluated by the Newcastle Ottawa scale^[8]^ in non-randomized trials.

#### 2.5.3 Heterogeneity assessment

We analyzed the subgroups determined in advance after checking the extracted data again and compared the efficacy of different subgroups directly. When heterogeneity was not easy to explain, it was included in the random effects model and I^2^ test was conducted to evaluate the degree of heterogeneity between studies.

### 2.6 Data synthesis and analysis

In this study, the mean difference and 95% Confidence interval (CI) were used as the efficacy analysis statistics for the included measurement data (continuous variables), and the odds ratio (OR) value and 95%CI were used as the efficacy analysis statistics for the included count data (2 category variables). Since “cured”, “markedly effective”, “effective”, and “ineffective” are the 4 levels of evaluation of curative effect generally recognized by the state or committee, and meta-analysis is a binary variable data, therefore “cure”, “significant effect”, and “effective” were combined into “effective”. Subgroup analysis was carried out according to the possible heterogeneity factors, such as different research types, different measurement indicators, different medication time, etc; when the included data among the subgroups were sufficiently similar (*P* > .1, I^2^ < 50%), the fixed response model was used for combined analysis; the random effect model was conducted for combined analysis if the studies within the subgroups had clinical homogeneity, but there was statistical heterogeneity; the sensitivity analysis was used when the heterogeneity was originated from the low-quality research; the qualitative description and analysis were carried out for the data that could not be combined; the funnel plot analysis was used to analyze the publication bias. Revman5.3 software (Copenhagen: The Nordic Cochrane Centre, The Cochrane Collaboration, 2014) provided by Cochrane Collaboration Network was used for meta-analysis; R software (R version 4.1.1 – "Kick Things"Copyright (C) 2021 The R Foundation for Statistical Computing.)was used for network meta-analysis; and Begg test was analyzed by using STATA14.0 software (Stata/SE 14.0 for Windows [64-bit x86-64].Revision 22Apr 2015.Copyright 1985-2015 StataCorp LP.).

## 3 Results

### 3.1 Literature search results

**Figure 1:**
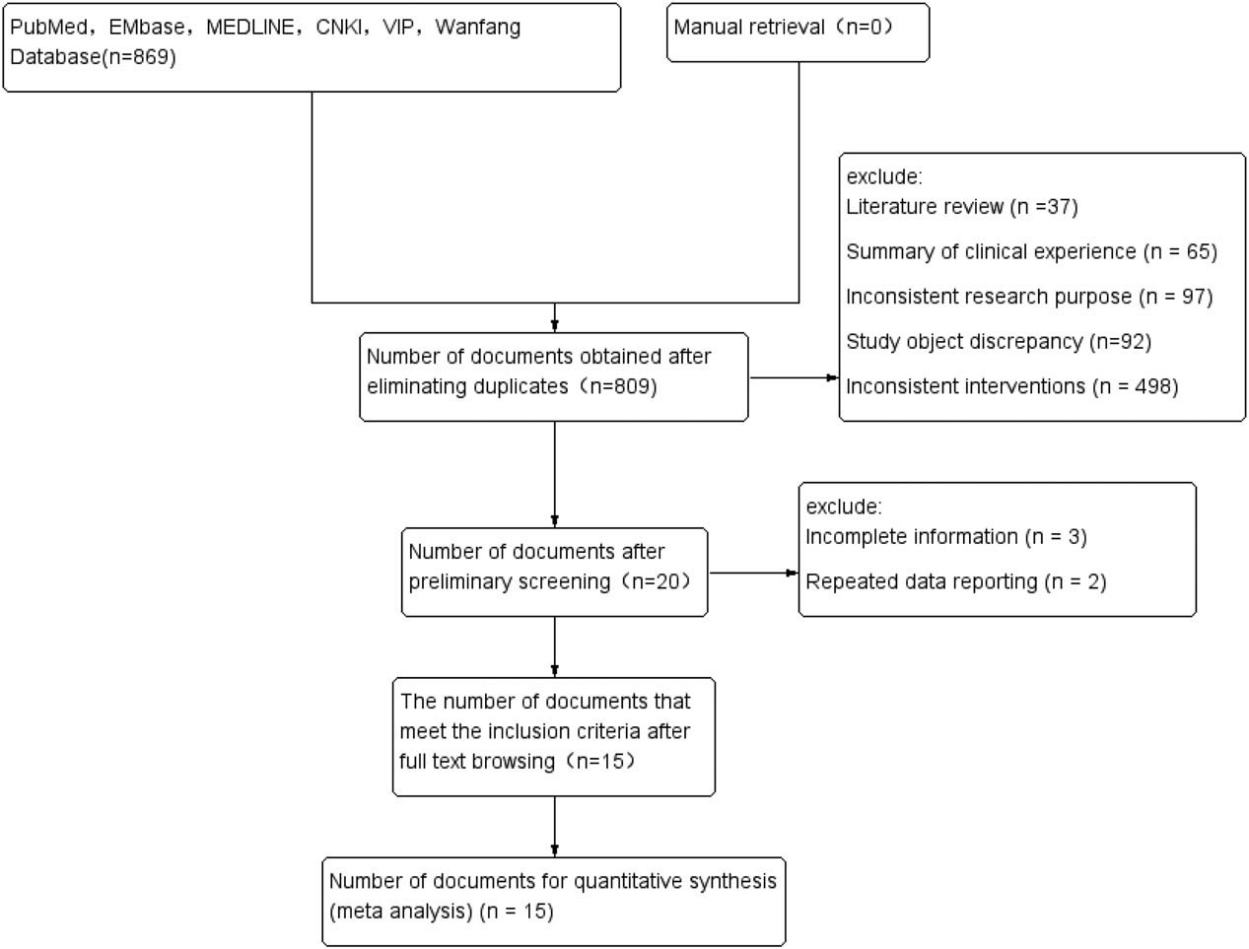
PRISMA literature screening flow chart.

Eight hundred sixty-nine related literatures were initially detected in 6 databases. Fifteen studies^[9–23]^ were included after step by step screening, with 1 English literature and 14 Chinese literature, and with a total of 1623 patients, including 854 patients in the experimental group and 769 patients in the control group. According to the requirements of PRISMA statement,^[24]^ a literature screening flow chart was developed, as shown in Figure 1. The basic characteristics of the included studies are shown in Tables 2 and 3.

**Table 2:** Basic features of the study (1).

		Sample (n)		Gender (male/female) (n)		Age (year)		Average course of disease (day)	
Studies	Year	T	C	T	C	T	C	T	C
Duan et al^[9]^	2020.3	82	41	39/43	23/18	51.99 ± 13.88	50.29 ± 13.17	2.71 ± 1.55	2.46 ± 1.49
Sun et al^[10]^	2020.7	32	25	17/15	11/14	45.4 ± 14.10	42.0 ± 11.70	11.7 ± 4.24	13.0 ± 10.5
Yu et al^[11]^	2020.4	147	148	82/65	89/59	48.27 ± 9.56	47.25 ± 8.67	–	–
Qiu et al^[12]^	2020.5	25	25	13/12	14/11	53.35 ± 18.35	51.32 ± 14.62	2.82 ± 0.79	3.21 ± 1.25
Fu et al^[13]^	2020.6	32	33	17/15	19/14	43. 26 ± 7. 15	43. 68 ± 6. 45	7. 56 ± 1. 25	8. 47 ± 1. 35
Fu et al^[14]^	2020.5	37	36	19/18	19/17	45.26 ± 7.25	44.68 ± 7.45	7.56 ± 1.25	8.47 ± 1.35
Hu et al^[15]^	2020.5	142	142	79/63	71/71	50.4 ± 15.2	51.8 ± 14.8	9.5 ± 5.1	9.9 ± 5.9
Cheng et al^[16]^	2020.3	51	51	26/25	27/24	55.5 ± 12.3	55.8 ± 11.6	≧6	≧6
Lu et al^[17]^	2020.4	63	38	28/35	18/20	59. 12 ± 16. 56	60. 20 ± 17. 01	–	–
Yang et al^[18]^	2020.7	26	23	16/10	9/14	50. 35 ± 13. 37	47. 17 ± 16. 57	–	–
Yao et al^[19]^	2020.2	21	21	16/5	12/9	57.1 ± 14.0	62.4 ± 12.3	12.8 ± 3.8	12.9 ± 3.3
Xiao et al^[20]^	2020.3	100	100	64/36	66/34	60.90 ± 8.70	62.20 ± 7.50	5.46 ± 2.09	6.37 ± 3.01
Qu et al^[21]^	2020.3	40	30	25/15	16/14	40.65 ± 8.23	39.82 ± 6.40	–	–
Zhang et al^[22]^	2020.4	22	22	10/12	12/10	49.05 ± 14.19	45.95 ± 14.68	3.27 ± 2.05	3.50 ± 2.16
Chen et al^[23]^	2020.6	34	34	14/20	15/19	65.06 ± 10.63	64.35 ± 10.34	14.68 ± 7.37	14.62 ± 6.34

C = the control group, T = the treatment group.

**Table 3:**
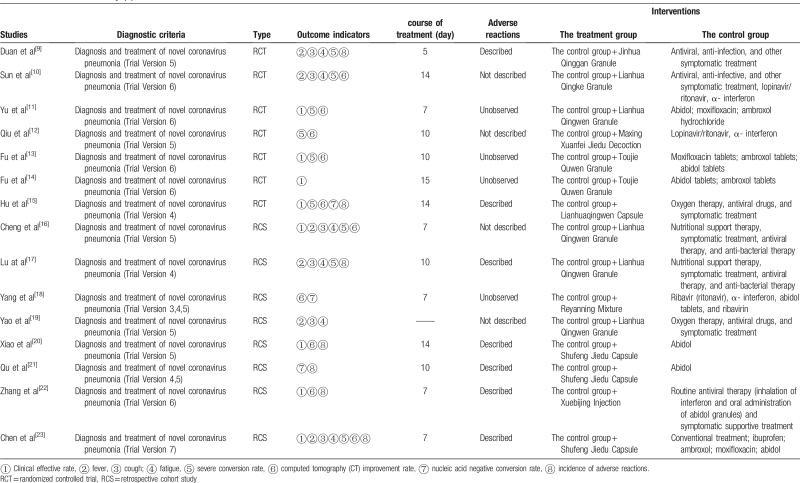
Basic features of the study (2).

### 3.2 Quality evaluation

All of the included 7 RCTs studies were conducted in China. They all mentioned the use of random grouping method, and did not describe the specific allocation concealment method. They all described that the blind method was not used and the baseline data of the 2 groups were comparable. There were no incomplete data reports and missing data. The treatment methods and outcome indicators of the treatment group and the control group were described in detail. Seven RCT original studies were evaluated by modified Jadad scale,^[7]^ including 2 articles with 4 points ,^[9,15]^ 4 articles with 3 points,^[10–13]^ and 1 article with 2 points.^[14]^ There were 1 low quality literature and 6 medium quality literature. The results of methodological quality evaluation are shown in Table 4.

**Table 4:** Quality evaluation of original research.

Studies	Randomization method	Allocation concealment	Blind method	Loss to follow-up	Baseline comparability	Jadad score
Duan et al^[9]^	Random number table by SPSS	Not described	Not used	Described	No significant difference	4
Sun et al^[10]^	Random number table method	Not described	Not used	Non	No significant difference	3
Yu et al^[11]^	Random number table method	Not described	Not used	Non	No significant difference	3
Qiu et al^[12]^	Random number table method	Not described	Not used	Non	No significant difference	3
Fu et al^[13]^	Random number table method	Not described	Not used	Non	No significant difference	3
Fu et al^[14]^	Random	Not described	Not used	Non	No significant difference	2
Hu et al^[15]^	Computer generated 1:1 grouping random scheme	Not described	Not used	Non	No significant difference	4

### 3.3 Quality evaluation of “Cochrane risk bias assessment tool”

**Figure 2:**
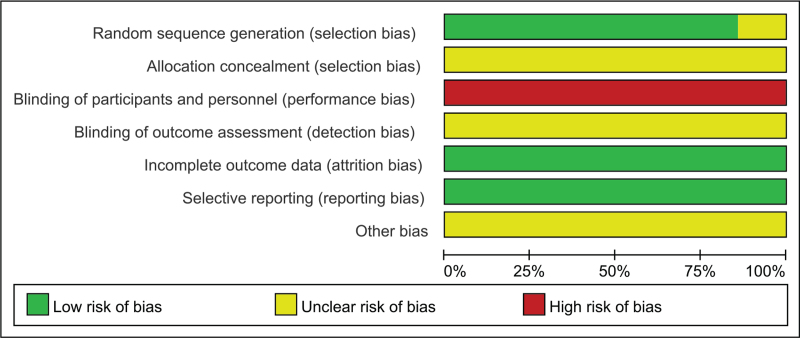
Bias risk percentage.

**Figure 3:**
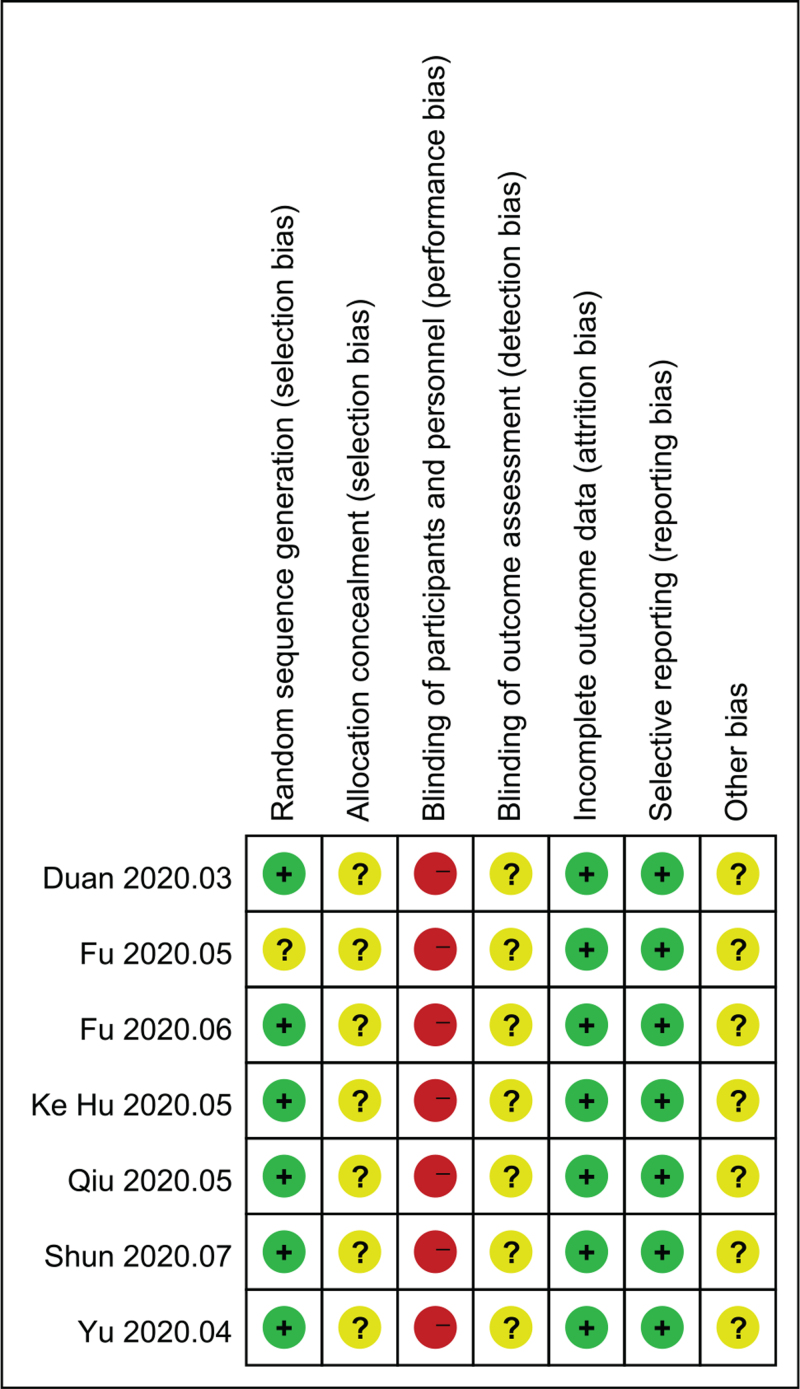
Bias risk summary chart.

The results showed that the low-risk proportion of random sequence generation in the selection bias of the original RCT study was about 41%, the moderate risk was about 45%, and the high risk was about 14% (see Fig. 2). By analogy, we know that there are certain bias in selection, implementation, and measurement of the included studies. The statistics of the bias of each literature is shown in Figure 3.

### 3.4 Quality evaluation of non-randomized control trials

Eight NRIs in this study were evaluated by Newcastle Ottawa scale. All NRIs results were moderate, indicating that the risk of bias was moderate. The results are shown in Table 5.

**Table 5:** Newcastle Ottawa scale (NOS).

	Select				Comparability^a^	Expose			
Studies	1	2	3	4	5	6	7	8	Total score^b^
	I	II	III	IV	V	VI	VII	VIII	
Cheng et al^[16]^	☆	☆	☆	☆	☆				5☆
Lu et al^[17]^	☆	☆	☆	☆	☆				5☆
Yang et al^[18]^	☆	☆	☆	☆	☆				5☆
Yao et al^[19]^	☆	☆	☆	☆	☆				5☆
Xiao et al^[20]^	☆	☆	☆	☆	☆				5☆
Qu et al^[21]^	☆	☆	☆	☆	☆				5☆
Zhang et al^[22]^	☆	☆	☆	☆	☆				5☆
Chen et al^[23]^	☆	☆	☆	☆	☆				5☆

a. Two stars with the highest comparability; b. Full score is 9; ☆. 1–8: Case-control studies (CC); I–VIII: Cohort studies (CS). 1. Case definition; 2. Case manifestations; 3. Selection of control group; 4. Definition of control group; 5: Choose the most important/second most important factor; 6. Determination of exposure; 7. Methods for determining cases and control groups; 8: No response rate. I. Representativeness of exposure; II. Selection of non-exposed persons; III. Determination of exposure; IV. Proof of no interesting results at the beginning; V. Comparability; VI. Evaluation of results; VII. Long enough follow-up time; VIII. Adequacy of follow-up.

### 3.5 Meta-analysis

#### 3.5.1 Primary outcomes: clinical effective rate

**Figure 4:**
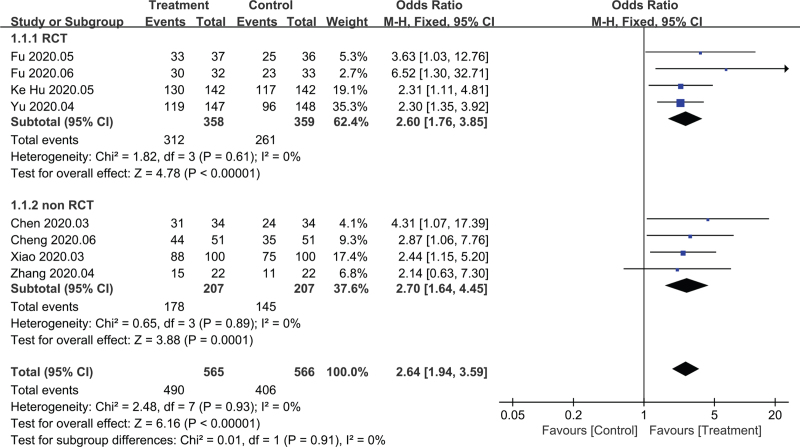
Clinical efficiency forest map.

Among the included studies, 8 trials^[11,13–15,16,20,22–23]^ reported clinical effective rate. The effective cases in treatment group and control group were 490 and 406, respectively. The fixed effects model was adopted for merger analysis because there was no statistical heterogeneity among the studies (*P* = .93, I^2^ = 0%). And the results showed that the clinical effective rate of the treatment group was higher than that of the control group (OR = 2.64, 95%CI [1.94,3.59], *P* < .00001). Subgroup analysis was done for RCT and non-RCT tests, respectively, and there was no significant heterogeneity between the 2 subgroups (*P* = .91, I^2^ = 0%). There was no statistical heterogeneity among the studies after the combination of RCT test (*P* = .61, I^2^ = 0%). The fixed effects model was conducted for merger analysis and the result showed that the clinical effective rate of treatment group was higher than that of control group (OR = 2.60, 95%CI [1.76,3.85], *P* < .00001). There was no statistical heterogeneity among the studies after the combination of non-RCT trials (*P* = .89, I^2^ = 0%). The fixed effects model was used for merger analysis and the results showed that the clinical effective rate of the treatment was higher than that of the control group (OR = 2.70, 95%CI [1.64,4.45], *P* < .0001). The results are shown in Figure 4.

#### 3.5.2 Secondary outcomes

Improvement rate of main symptoms; disappearance time of main symptoms; improvement rate of CT; severe conversion rate; negative conversion rate of nucleic acid; and adverse reactions.

Meta-analysis was carried out on the data of 15 studies in which the same index was scored by the same or similar scoring method. The results of heterogeneity test, model, effect amount, and effect value are shown in Table 6. The treatment group was better than the control group in improving the symptoms of fever, cough, and fatigue, shortening the treatment time of patients with fever, cough, and fatigue symptoms, improving the CT improvement rate of patients and reducing the conversion rate of patients to severe cases. However, there was no significant difference between the treatment group and the control group in improving the negative rate of nucleic acid and reducing the incidence of adverse reactions different.

**Table 6:** Meta-analysis results of secondary outcomes.

		Heterogeneity test		Effective number of cases					
Outcomes	Type	Qualitative test for *P* values	Quantitative test for *I*^2^ (%)	The treatment group (n)	The control group (n)	Model	Effect quantity	Effect value and 95%CI	Effect *P* value
Improvement rate of main symptoms	Fever (RCT)	–	–	57	22	Fixed	OR	3.60[1.43,9.04]	.006
	Fever (non-RCT)	.87	0	124	75	Fixed	OR	3.71[1.97,7.00]	<.0001
	Fever (total)	.95	0	181	97	Fixed	OR	3.68[2.18,6.20]	<.00001
	Cough (RCT)	.4	0	70	28	Fixed	OR	3.26[1.52,7.00]	.002
	Cough (non-RCT)	.37	4	81	39	Fixed	OR	3.87[2.20,6.82]	<.00001
	Cough (total)	.56	0	151	67	Fixed	OR	3.65[2.32,5.74]	<.00001
	Fatigue (RCT)	.53	0	59	22	Fixed	OR	3.36[1.32,8.52]	.01
	Fatigue (non-RCT)	.6	0	75	45	Fixed	OR	3.25[1.71,6.21]	.0003
	Fatigue (total)	.81	0	134	67	Fixed	OR	3.29[1.93,5.59]	<.0001
Disappearance time of main symptoms	Fever (RCT)	–	–	25	25	Random	MD	–1.68[–2.38,–0.98]	<.00001
	Fever (non-RCT)	.06	56	215	195	Random	MD	–1.27[–1.80,–0.75]	<.00001
	Fever (total)	.05	55	240	220	Random	MD	–1.35[–1.80,–0.90]	<.00001
	Cough (RCT)	–	–	25	25	Random	MD	–2.93[–4.02,–1.84]	<.00001
	Cough (non-RCT)	<.0001	88	186	168	Random	MD	–1.27[–2.56,0.02]	.05
	Cough (total)	<.00001	88	211	193	Random	MD	–1.35[–1.80,–0.90]	.01
	Fatigue (non-RCT)	<.00001	90	177	159	Random	MD	–1.27[–2.20,–0.33]	.008
CT improvement rate	RCT	.11	46	283	224	Fixed	OR	2.11[1.51,2.94]	<.0001
	Non-RCT	.43	0	190	151	Fixed	OR	2.48[1.58,3.91]	<.0001
	Total	.24	22	473	375	Fixed	OR	2.23[1.71,2.92]	<.00001
Severe conversion rate	RCT	.96	0	34	57	Fixed	OR	0.46[0.29,0.73]	.001
	Non-RCT	.96	0	11	26	Fixed	OR	0.31[0.15,0.66]	.002
	Total	.98	0	45	83	Fixed	OR	0.41[0.28,0.61]	<.0001
Negative conversion rate of nucleic acid	RCT	–	–	109	101	Random	OR	1.34[0.79,2.28]	.28
	Non-RCT	.04	75	37	21	Random	OR	3.97[0.36,43.87]	.26
	Total	.09	59	146	122	Random	OR	2.00[0.74,5.39]	.17
Incidence of adverse reactions	RCT	.002	90	92	77	Random	OR	4.47[0.05,410.62]	.52
	Non-RCT	.33	13	9	17	Random	OR	0.44[0.17,1.14]	.09
	Total	.02	62	101	94	Random	OR	0.77[0.27,2.17]	.62

CT = computer tomography, MD = mean difference, OR = odds ratio, RCT = randomized controlled trial.

#### 3.5.3 Publication bias analysis

**Figure 5:**
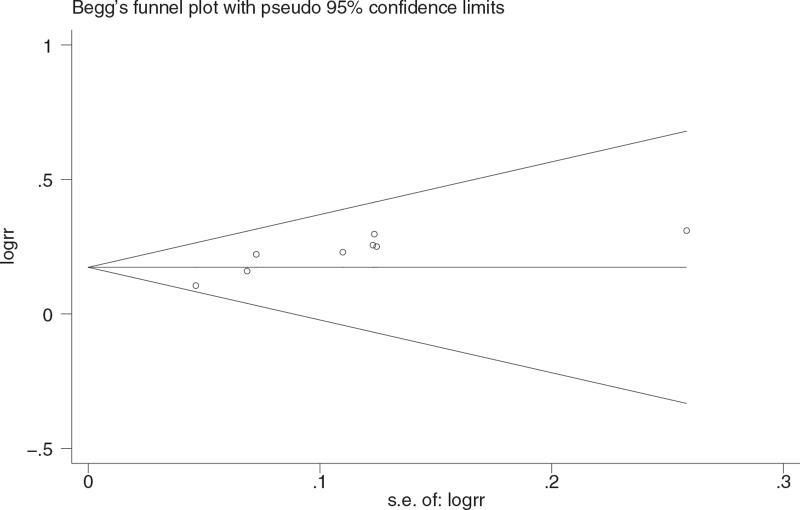
The funnel chart of bias generation detected by Begg rank correlation.

Begg test was used to detect the bias of the statistical results of clinical efficiency. The results showed that $\mathrm{Pr}>\left\lceil \text{z}\right\rceil =0.266$, indicating that there was no obvious bias in this study. The results are shown in Table 7 and Figure 5.

**Table 7:** Detection results of bias in the study by Begg test.

Begg test	
Adj. Kendall score (P-Q)	10
Std. dev. of score	8.08
Number of studies	8
z	1.24
Pr > |z|	0.216
z	1.11 (continuity corrected)
Pr > |z|	0.266 (continuity corrected)

#### 3.5.4 Sensitivity analysis

**Figure 6:**
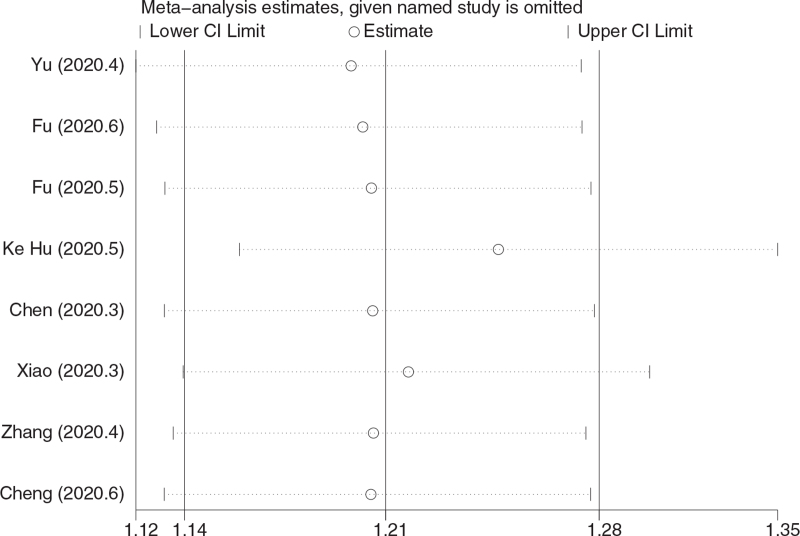
Sensitivity analysis results of clinical efficacy.

Sensitivity analysis of the results of the clinical efficacy study showed that the lowest limit of all the study results was not lower than the number 1, indicating that there was no significant difference in the results after removing any study. It is proved that the sensitivity of clinical effective quantity is low, and it has good stability, reliability, and the analysis result is stable and credible. See Table 8 and Figure 6.

**Table 8:** Sensitivity analysis results data of clinical efficacy.

Study omitted	Estimate	95%CI	
Yu (2020.4)	1.1968488	1.1211154	1.2776979
Fu (2020.6)	1.2008638	1.1284314	1.2779458
Fu (2020.5)	1.2038916	1.1313183	1.2811203
Ke Hu (2020.5)	1.2485584	1.1575173	1.3467603
Chen (2020.3)	1.2043625	1.1311516	1.282312
Xiao (2020.3)	1.2169871	1.1377463	1.3017468
Zhang (2020.4)	1.204604	1.1342237	1.2793516
Cheng (2020.6)	1.2037128	1.1310836	1.2810057
Combined	1.2089164	1.1382247	1.2839984

### 3.6 Network meta-analysis

#### 3.6.1 Clinical effective rate

**Figure 7:**
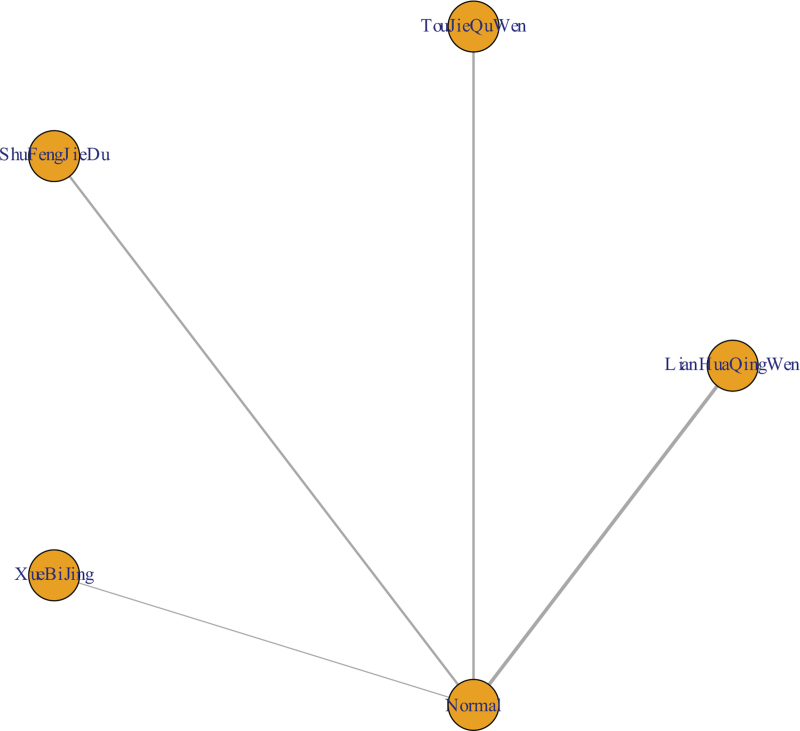
Network evidence map of network meta-analysis.

**Figure 8:**
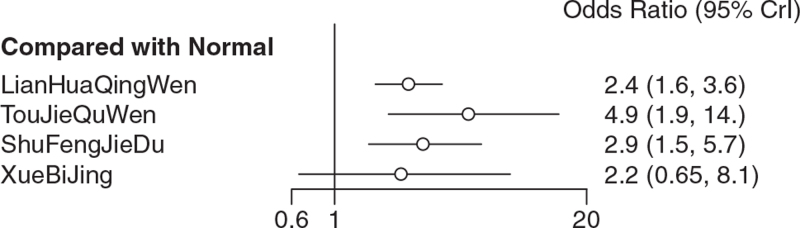
Meta-analysis on clinical effective rate of 4 kinds of traditional Chinese Medicine.

**Figure 9:**
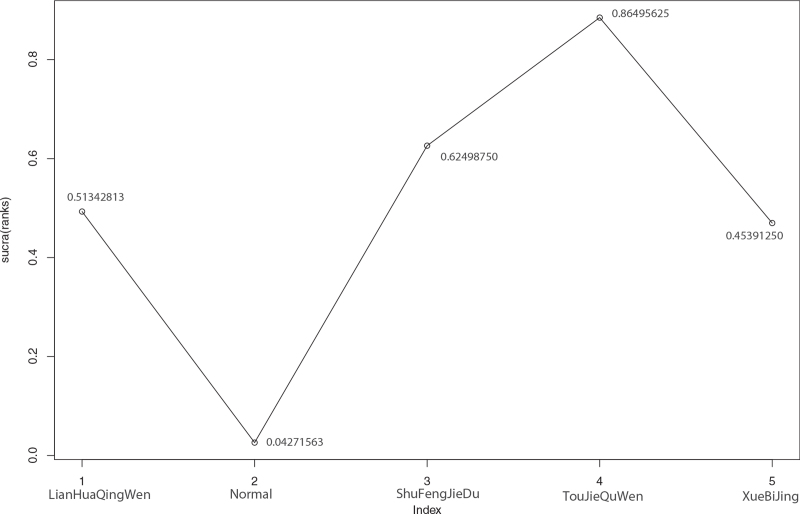
Sucra (rank) of clinical effective rate of 4 kinds of traditional Chinese Medicine. 1. LianHuaQingWen (sucra [rank] = 0.51342813), 2. Normal (sucra [rank] = 0.04271563), 3. ShuFengJieDu (sucra [rank] = 0.62498750), 4. TouJieQuWen (sucra [rank] = 0.86495625), 5. XueBiJing (sucra [rank] = 0.45391250).

Among the 15 literatures included, 8 reported the effective rate of TCM in the treatment of COVID-19, of which Xuebijing treated COVID-19 in 1 case,^[22]^ Shufeng Jiedu treatment in 2 cases,^[20,23]^ Toujie Quwen treatment in 2 cases,^[13,14]^ and Lianhua Qingwen in 3 cases,^[11,15–16]^ as shown in Figure 7. According to the results of R software network meta-analysis, the effective rates of Shufeng Jiedu (OR = 2.9, 95%CI [1.5,5.7]), Toujie Quwen (OR = 4.9, 95%CI [1.9,14.0]), and Lianhua Qingwen (OR = 2.4, 95%CI [1.6,3.6]) were better than those of the conventional treatment group, the difference was statistically significant, and there was no effective rate difference between the 4 TCMs. In terms of the ranking order, the effective rates are as follows: Toujie Quwen > Shufeng Jiedu > Lianhua Qingwen > Xuebijing > Normal (conventional treatment). See Figures 8 and 9 and Table 9 for details.

**Table 9:** Network meta-analysis on clinical effective rate of 4 kinds of traditional Chinese medicine.

LianHuaQingWen	**0.41 (0.20, 0.78)**	1.18 (0.40, 3.79)	2.00 (0.57, 8.05)	0.91 (0.18, 4.94)
**2.43 (1.59, 3.62)**	Normal	**2.91 (1.50, 5.71)**	**4.94 (1.92, 14.20)**	2.25 (0.52, 10.28)
0.85 (0.26, 2.47)	**0.34 (0.13, 0.84)**	ShuFengJieDu	1.66 (0.40, 8.05)	0.76 (0.13, 4.47)
0.50 (0.12, 1.74)	**0.20 (0.06, 0.58)**	0.60 (0.12, 2.50)	TouJieQuWen	0.44 (0.07, 2.92)
1.10 (0.20, 5.53)	0.44 (0.10, 1.93)	1.32 (0.22, 7.53)	2.26 (0.34, 14.70)	XueBiJing

#### 3.6.2 Main clinical symptoms

##### 3.6.2.1 Fever

**Figure 10:**
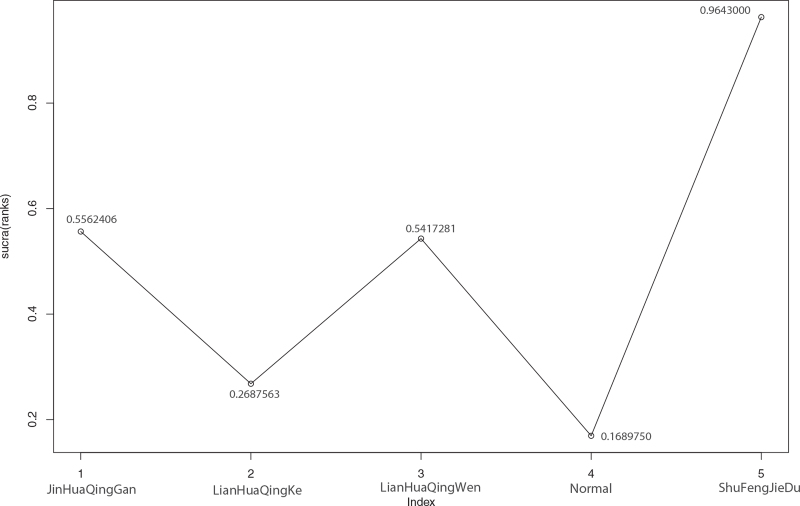
Sucra (rank) of fever effective rate of 4 kinds of traditional Chinese Medicine. 1. JinHuaQingGan (sucra [rank] = 0.5562406), 2. LianHuaQingKe (sucra [rank] = 0.2687563), 3. LianHuaQingWen (sucra [rank] = 0.5417281), 4. Normal (sucra [rank] = 0.1689750), 5. ShuFengJieDu (sucra [rank] = 0.9643000).

Among the 15 included literatures, 6 reported the effective rate of TCM in the treatment of COVID-19, of which Jinhua Qinggan treated COVID-19 in 1 case,^[9]^ Lianhua Qingke in 1 case,^[10]^ Lianhua Qingwen in 3 cases,^[16–17,19]^ and Shufeng Jiedu in 1 case.^[23]^ According to the results of R software network meta-analysis, Jinhua Qinggan (OR = –1.3, 95%CI [–2.27,–0.38]), Lianhua Qingwen (OR = –1.26, 95%CI [–1.94,–0.6]), and Shufeng Jiedu (OR = –17.57, 95%CI [–52.31,–2.52]) were better than conventional treatment group in the effective rate; and among the 4, Shufeng Jiedu was superior to Jinhua Qinggan (OR = –16.27, 95%CI [–51.05,–1.15]) and Lianhua Qingwen (OR = –16.32, 95%CI [–51.04,–1.23]); in terms of the effective rate of the 4 kinds of TCM, they rank as follows: Shufeng Jiedu > Jinhua Qinggan > Lianhua Qingwen > Lianhua Qingke > Normal (conventional treatment), and Shufeng Jiedu was the best. See Table 10 and Figure 10 for details.

**Table 10:** Network meta-analysis on fever effective rate of 4 kinds of traditional Chinese Medicine.

JinHuaQingGan	–12.87 (–74.92, 36.77)	–0.04 (–1.2, 1.11)	**16.27 (1.15, 51.05)**	**–1.3 (–2.27, –0.38)**
12.87 (–36.77, 74.92)	LianHuaQingKe	12.83 (–36.87, 74.91)	32.41 (–22.46, 100.6)	11.58 (–38.17, 73.63)
0.04 (–1.11, 1.2)	–12.83 (–74.91, 36.87)	LianHuaQingWen	**16.32 (1.23, 51.04)**	**–1.26 (–1.94, –0.6)**
**–16.27 (–51.05, –1.15)**	–32.41 (–100.6, 22.46)	**–16.32 (–51.04, –1.23)**	ShuFengJieDu	**–17.57 (–52.31, –2.52)**
1.3 (0.38, 2.27)	–11.58 (–73.63, 38.17)	1.26 (0.6, 1.94)	**17.57 (2.52, 52.31)**	Normal

##### 3.6.2.2 Cough

**Figure 11:**
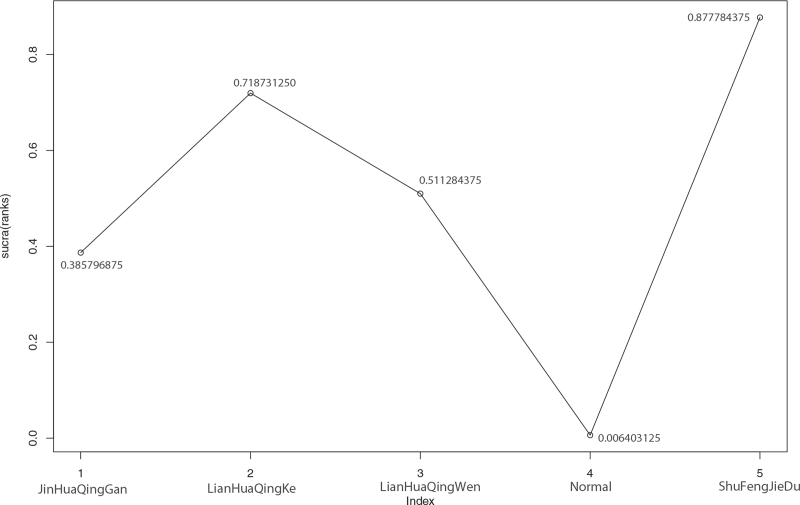
Sucra (rank) of cough response rate of 4 kinds of traditional Chinese Medicine. 1. JinHuaQingGan (sucra [rank] = 0.385796875), 2. LianHuaQingKe (sucra [rank] = 0.718731250), 3. LianHuaQingWen (sucra [rank] = 0.511284375), 4. Normal (sucra [rank] = 0.006403125), 5. ShuFengJieDu (sucra [rank] = 0.877784375).

Among the 15 included literatures, 6 reported the effective rate of TCM in the treatment of cough symptoms of COVID-19, including Jinhua Qinggan in the treatment of COVID-19 in 1 case,^[9]^ Lianhua Qingke in 1 case,^[10]^ Lianhua Qingwen in 3 cases,^[16–17,19]^ and Shufeng Jiedu in 1 case.^[23]^ According to the results of R software network meta-analysis, Jinhua Qinggan (OR = –0.97, 95%CI [–1.91,–0.06]), Lianhua Qingke (OR = –1.79, 95%CI [–3.45,–0.39]), Lianhua Qingwen (OR = –1.25, 95%CI [–1.87,–0.64]), and Shufeng Jiedu (OR = –2.36, 95%CI [–4.44,–0.79]) were better than those of the conventional treatment group. The effective rates of Lianhua Qingke (OR = –0.82, 95%CI [–2.71,0.88]), Lianhua Qingwen (OR = –0.28, 95%CI [–1.37, 0.85]), and Shufeng Jiedu (OR = –1.39, 95%CI [–3.67,0.45]) were better than Jinhua Qinggan. The effective rates of Lianhua Qingke (OR = –0.54, 95%CI [–2.31,0.997]) and Shufeng Jiedu (OR = –1.11, 95%CI [–3.28,0.59]) were better than Lianhua Qingwen. According to the rank order, Shufeng Jiedu > Lianhua Qingke > Lianhua Qingwen > Jinhua Qinggan > Normal (conventional treatment), Shufeng Jiedu and Lianhua Qingke had the best efficacy. See Table 11 and Figure 11 for details.

**Table 11:** Network meta-analysis on cough response rate of 4 kinds of traditional Chinese medicine.

JinHuaQingGan	0.82 (–0.88, 2.71)	0.28 (–0.85, 1.37)	1.39 (–0.45, 3.67)	**–0.97 (–1.91, –0.06)**
**–0.82 (–2.71, 0.88)**	LianHuaQingKe	**–0.54 (–2.31, 0.997)**	0.58 (–1.69, 3.06)	**–1.79 (–3.45, –0.39)**
**–0.28 (–1.37, 0.85)**	0.54 (–0.997, 2.31)	LianHuaQingWen	1.11 (–0.59, 3.28)	**–1.25 (–1.87, –0.64)**
**–1.39 (–3.67, 0.45)**	–0.58 (–3.06, 1.69)	**–1.11 (–3.28, 0.59)**	ShuFengJieDu	**–2.36 (–4.44, –0.79)**
0.97 (0.06, 1.91)	1.79 (0.39, 3.45)	1.25 (0.64, 1.87)	2.36 (0.79, 4.44)	Normal

##### 3.6.2.3 Fatigue

**Figure 12:**
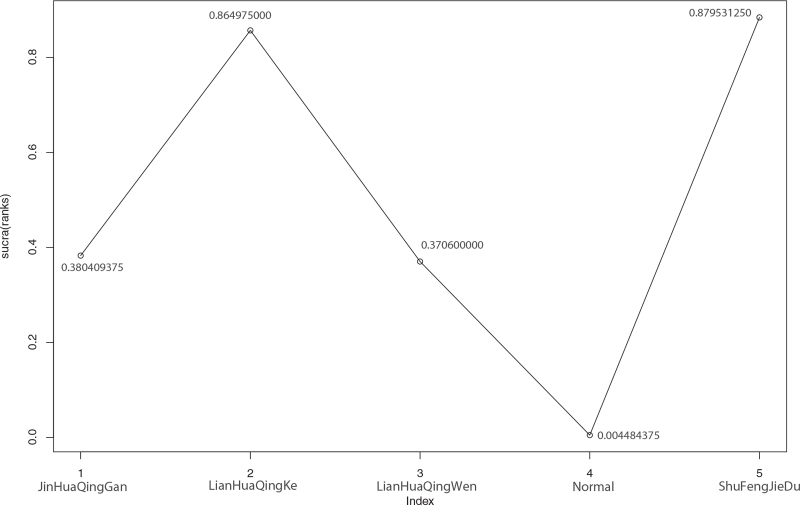
Sucra (rank) of fatigue and effective rate of 4 kinds of traditional Chinese Medicine. 1. JinHuaQingGan (sucra [rank] = 0.380409375), 2. LianHuaQingKe (sucra [rank] = 0.864975000), 3. LianHuaQingWen (sucra [rank] = 0.370600000), 4. Normal (sucra [rank] = 0.004484375), 5. ShuFengJieDu (sucra [rank] = 0.879531250).

Among the 15 included literatures, 6 reported the effective rate of TCM in the treatment of fatigue symptoms of COVID-19, among which Jinhua Qinggan treated COVID-19 in 1 case,^[9]^ Lianhua Qingke in 1 case,^[10]^ Lianhua Qingwen in 3 cases,^[16–17,19]^ and Shufeng Jiedu in 1 case.^[23]^ According to the results of R software network meta-analysis, Jinhua Qinggan (OR = –1.11, 95%CI [–2.12,–0.11]), Lianhua Qingke (OR = –21.89, 95%CI [–60.45,–2.54]), Lianhua Qingwen (OR = –1.07, 95%CI [–1.77,–0.38]), and Shufeng Jiedu (OR = –23.55, 95%CI [–61.99,–3.06]) were better than those of the conventional treatment group, and the difference was statistically significant. The effective rates of Lianhua Qingke (OR = –20.77, 95%CI [–59.34,–1.37]) and Shufeng Jiedu (OR = –22.43, 95%CI [–60.84,–1.91]) were better than Jinhua Qinggan. The effective rates of Lianhua Qingke (OR = –20.84, 95%CI [–59.41,–1.42]) and Shufeng Jiedu (OR = –22.43, 95%CI [–60.94,–1.97]) were better than those of Lianhua Qingwen. According to the rank order, Shufeng Jiedu > Lianhua Qingke > Jinhua Qinggan > Lianhua Qingwen > Normal (conventional treatment), Shufeng Jiedu and Lianhua Qingke had the best efficacy. See Table 12 and Figure 12 for details.

**Table 12:** Network meta-analysis on fatigue efficiency of 4 kinds of traditional Chinese medicine.

JinHuaQingGan	**20.77 (1.37, 59.34)**	–0.04 (–1.27, 1.17)	**22.43 (1.91, 60.84)**	**–1.11 (–2.12, –0.11)**
**–20.77 (–59.34, –1.37)**	LianHuaQingKe	**–20.84 (–59.41, –1.42)**	1.52 (–45.35, 47.06)	**–21.89 (–60.45, –2.54)**
0.04 (–1.17, 1.27)	**20.84 (1.42, 59.41)**	LianHuaQingWen	**22.43 (1.97, 60.94)**	**–1.07 (–1.77, –0.38)**
**–22.43 (–60.84, –1.91)**	–1.52 (–47.06, 45.35)	**–22.43 (–60.94, –1.97)**	ShuFengJieDu	**–23.55 (–61.99, –3.06)**
1.11 (0.11, 2.12)	**21.89 (2.54, 60.45)**	1.07 (0.38, 1.77)	**23.55 (3.06, 61.99)**	Normal

#### 3.6.3 CT improvement rate

**Figure 13:**
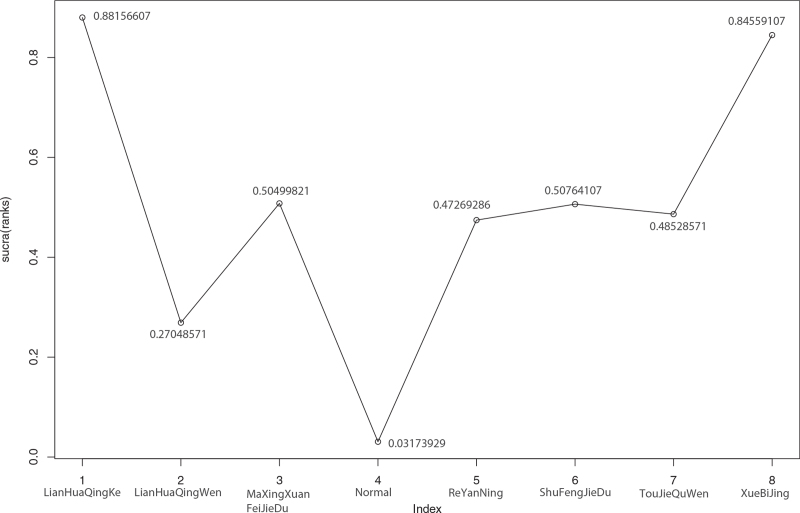
Sucra (rank) of CT improvement rate of 7 kinds of traditional Chinese Medicine. 1. LianHuaQingKe (sucra [rank] = 0.88156607), 2. LianHuaQingWen (sucra [rank] = 0.27048571), 3. MaXingXuanFeiJieDu (sucra [rank] = 0.50499821), 4. Normal (sucra (rank) = 0.03173929), 5. ReYanNing (sucra [rank] = 0.47269286), 6. ShuFengJieDu (sucra [rank] = 0.50764107), 7. TouJieQuWen (sucra [rank] = 0.48528571), 8. XueBiJing (sucra [rank] = 0.84559107). CT = computed tomography.

Among the 15 included articles, 10 reported the improvement rate of CT in patients with COVID-19 by TCM. Among them, Lianhua Qingke treated COVID-19 in 1 case,^[10]^ Lianhua Qingwen in 3 cases,^[11,15–16]^ Maxing Xuanfeijiedu in 1 case,^[12]^ Reyanning in 1 case,^[18]^ Shufeng Jiedu in 2 cases,^[20,23]^ Toujie Quwen in 1 case,^[13]^ and Xuebijing in 1 case.^[22]^ According to the results of R software network meta-analysis, the CT improvement rate of 7 kinds of TCM on patients with COVID-19 was better than that of conventional treatment group, the difference was statistically significant. The CT improvement rate of Lianhua Qingke was better than that of Lianhua Qingwen (OR = –2.25, 95%CI [–5.77,–0.2]), Toujie Quwen (OR = –1.78, 95%CI [–5.39,0.58]), Reyanning (OR = –1.8, 95%CI [–5.59,0.87]), and Shufeng Jiedu (OR = –1.75, 95%CI [–5.28,0.36]). The CT improvement rate of Xuebijing was better than that of Lianhua Qingwen (OR = –2.03, 95%CI [–5.45,0.03]), Toujie Quwen (OR = –1.56, 95%CI [–5.12,0.85]), and Shufeng Jiedu (OR = –1.54, 95%CI [–5.01,0.62]). In terms of ranking order, Lianhua Qingke > Xuebijing > Shufeng Jiedu > Maxing Xuanfeijiedu > Toujie Quwen > Reyanning > Lianhua Qingwen > Normal (conventional treatment), and the CT improvement rate of Lianhua Qingke and Xuebijing was the highest. See Table 13 and Figure 13 for details.

**Table 13:** NMA of CT improvement rate of 7 kinds of traditional Chinese medicine.

LianHuaQingKe	**–2.25 (–5.77, –0.2)**	–1.7 (–5.47, 1.28)	**–1.78 (–5.39, 0.58)**	**–1.8 (–5.59, 0.87)**	**–1.75 (–5.28, 0.36)**	–0.22 (–4.21, 3.73)	**–2.83 (–6.32, –0.84)**
2.25 (0.2, 5.77)	LianHuaQingWen	0.57 (–1.19, 2.75)	0.48 (–0.68, 1.74)	0.47 (–1.11, 2.24)	0.5 (–0.22, 1.23)	2.03 (–0.03, 5.45)	**–0.59 (–0.92, –0.26)**
1.7 (–1.28, 5.47)	–0.57 (–2.75, 1.19)	MaXingXuanFeiJieDu	–0.1 (–2.52, 2.04)	–0.11 (–2.73, 2.35)	–0.08 (–2.33, 1.79)	1.48 (–1.53, 5.21)	**–1.16 (–3.32, 0.57)**
1.78 (–0.58, 5.39)	**–0.48 (–1.74, 0.68)**	0.1 (–2.04, 2.52)	TouJieQuWen	–0.01 (–1.96, 2.05)	0.02 (–1.36, 1.32)	1.56 (–0.85, 5.12)	**–1.07 (–2.28, 0.05)**
1.8 (–0.87, 5.59)	–0.47 (–2.24, 1.11)	0.11 (–2.35, 2.73)	0.01 (–2.05, 1.96)	ReYanNing	0.03 (–1.79, 1.7)	1.59 (–1.13, 5.2)	**–1.06 (–2.8, 0.48)**
1.75 (–0.36, 5.28)	**–0.5 (–1.23, 0.22)**	0.08 (–1.79, 2.33)	–0.02 (–1.32, 1.36)	–0.03 (–1.7, 1.79)	ShuFengJieDu	1.54 (–0.62, 5.01)	**–1.09 (–1.75, –0.46)**
0.22 (–3.73, 4.21)	**–2.03 (–5.45, 0.03)**	–1.48 (–5.21, 1.53)	**–1.56 (–5.12, 0.85)**	–1.59 (–5.2, 1.13)	**–1.54 (–5.01, 0.62)**	XueBiJing	**–2.62 (–6.02, –0.59)**
2.83 (0.84, 6.32)	**0.59 (0.26, 0.92)**	1.16 (–0.57, 3.32)	1.07 (–0.05, 2.28)	1.06 (–0.48, 2.8)	1.09 (0.46, 1.75)	2.6 (0.59, 6.02)	Normal

CT = computed tomography.

#### 3.6.4 Severe conversion rate

**Figure 14:**
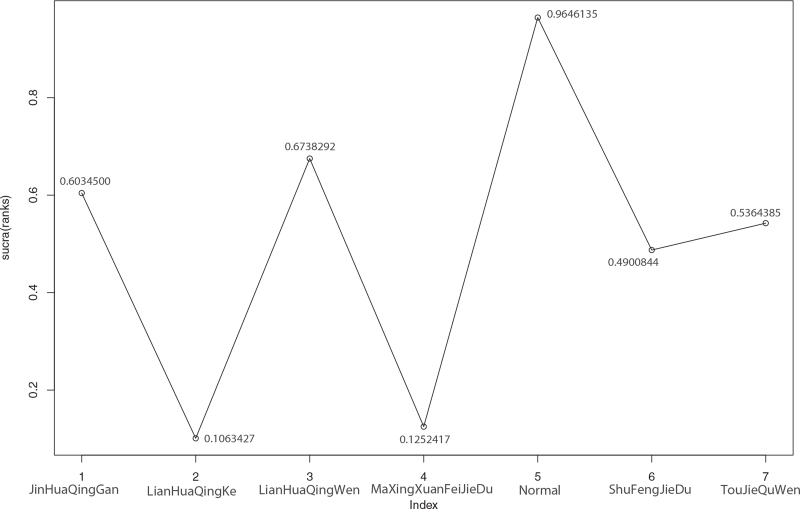
Sucra (rank) of severe conversion rate of 6 kinds of traditional Chinese Medicine. 1. JinHuaQingGan (sucra [rank] = 0.6034500), 2. LianHuaQingKe (sucra [rank] = 0.1063427), 3. LianHuaQingWen (sucra [rank] = 0.6738292), 4. MaXingXuanFeiJieDu (sucra [rank] = 0.1252417), 5. Normal (sucra [rank] = 0.9646135), 6. ShuFengJieDu (sucra [rank] = 0.4900844), 7. TouJieQuWen (sucra [rank] = 0.5364385).

Among the 15 included articles, 9 reported the severe conversion rate of diseases in patients with COVID-19 treated by TCM, including Jinhua Qinggan treating COVID-19 in 1 case,^[9]^ Lianhua Qingke in 1 case,^[10]^ Lianhua Qingwen in 4 cases,^[11,15–17]^ Maxing Xuanfeijiedu in 1 case,^[12]^ Shufeng Jiedu in 1 case,^[23]^ and Toujie Quwen in 1 case.^[13]^ According to the results of R software network meta-analysis, the severe conversion rate of the 6 kinds of TCM on patients with COVID-19 was better than that of the conventional treatment group, and the difference was statistically significant. The severe conversion rate of Lianhua Qingke was lower than that of Jinhua Qinggan (OR = –11.32, 95%CI [–37.8,–0.14]), Shufeng Jiedu (OR = –10.89, 95%CI [–37.28,0.43]), Toujie Quwen (OR = –10.83, 95%CI [–37.39,0.87]), and Lianhua Qingwen (OR = –11.52, 95%CI [–37.95,–0.4]). The severe conversion rate of Maxing Xuanfeijiedu (OR = –12.6, 95%CI [–46.96,0.95]) was lower than that of Lianhua Qingwen. In terms of ranking order, Normal (conventional treatment) > Lianhua Qingwen > Jinhua Qinggan > Toujie Quwen > Shufeng Jiedu > Maxing Xuanfeijiedu > Lianhua Qingke, and Lianhua Qingke had the lowest conversion rate. See Table 14 and Figure 14 for details.

**Table 14:** NMA of severe conversion rate of 6 kinds of traditional Chinese medicine.

JinHuaQingGan	**–11.32 (–37.8, –0.14)**	0.2 (–0.92, 1.33)	–12.39 (–46.68, 1.2)	–0.42 (–4.01, 2.11)	–0.43 (–2.35, 1.29)	0.974 (–0.04, 2.01)
11.32 (0.14, 37.8)	LianHuaQingKe	11.52 (0.4, 37.95)	–0.49 (–37.98, 28.17)	10.83 (–0.87, 37.39)	10.89 (–0.43, 37.28)	**12.29 (1.19, 38.73)**
**–0.2 (–1.33, 0.92)**	**–11.52 (–37.95, –0.4)**	LianHuaQingWen	**–12.6 (–46.96, 0.95)**	–0.6 (–4.1, 1.73)	**–0.62 (–2.33, 0.83)**	0.78 (0.31, 1.26)
12.39 (–1.2, 46.68)	0.49 (–28.17, 37.98)	12.6 (–0.95, 46.96)	MaXingXuanFeiJieDu	11.88 (–2.08, 46.34)	11.92 (–1.65, 46.34)	13.38 (–0.16, 47.69)
0.42 (–2.11, 4.01)	**–10.83 (–37.39, 0.87)**	0.6 (–1.73, 4.09)	–11.88 (–46.34, 2.08)	TouJieQuWen	–0.02 (–2.85, 3.67)	1.37 (–0.91, 4.84)
0.43 (–1.29, 2.35)	**–10.89 (–37.28, 0.43)**	0.62 (–0.83, 2.33)	–11.92 (–46.34, 1.65)	0.02 (–3.67, 2.85)	ShuFengJieDu	1.39 (0.04, 3.04)
**–0.97 (–2.01, 0.04)**	**–12.29 (–38.73, –1.19)**	**–0.78 (–1.26, –0.31)**	**–13.38 (–47.69, 0.16)**	**–1.37 (–4.84, 0.91)**	**–1.39 (–3.04, –0.04)**	Normal

#### 3.6.5 Negative conversion rate of nucleic acid

**Figure 15:**
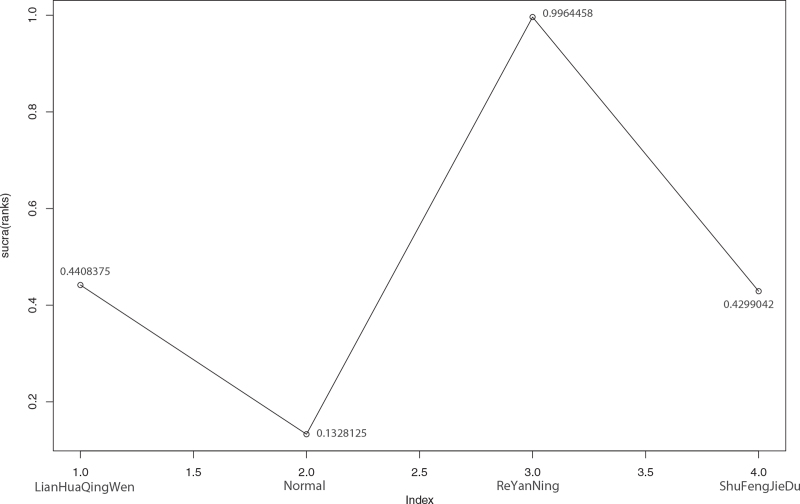
Sucra (rank) of nucleic acid negative conversion rate of 3 kinds of traditional Chinese Medicine. 1. LianHuaQingWen (sucra [rank] = 0.4408375), 2. Normal (sucra [rank] = 0.1328125), 3. ReYanNing (sucra [rank] = 0.9964458), 4. ShuFengJieDu (sucra [rank] = 0.4299042).

Among the 15 included articles, 3 reported the negative conversion rate of nucleic acid in the treatment of COVID-19 by TCM, among which 1 was treated by Lianhua Qingwen,^[15]^ 1 by Reyanning,^[18]^ and 1 by Shufeng Jiedu.^[21]^ According to the results of R software network meta-analysis, Lianhua Qingwen (OR = –0.3, 95%CI [–0.83,0.24]), Reyanning (OR = –3.14, 95%CI [–6.53, –1.17]), and Shufeng Jiedu (OR = –0.36, 95%CI [–1.5,0.73]) were better than those of the conventional treatment group, with statistically significant differences. The effective rate of Reyanning was better than Shufeng Jiedu (OR = –2.81, 95%CI [–6.33,–0.49]) and Lianhua Qingwen (OR = –2.85, 95%CI [–6.27,–0.79]). In terms of ranking order, Reyanning > Lianhua Qingwen > Shufeng Jiedu > Normal (conventional treatment), and Reyanning had the best negative conversion rate. See Table 15 and Figure 15 for details.

**Table 15:** NMA of nucleic acid negative conversion rate of 3 traditional Chinese medicines.

LianHuaQingWen	2.85 (0.79, 6.27)	0.06 (–1.15, 1.33)	**–0.3 (–0.83, 0.24)**
**–2.85 (–6.27, –0.79)**	ReYanNing	**–2.81 (–6.33, –0.49)**	**–3.14 (–6.53, –1.17)**
–0.06 (–1.33, 1.15)	2.81 (0.49, 6.33)	ShuFengJieDu	**–0.36 (–1.5, 0.73)**
**0.3 (–0.24, 0.83)**	**3.14 (1.17, 6.53)**	0.36 (–0.73, 1.5)	Normal

#### 3.6.6 Incidence of adverse reactions

**Figure 16:**
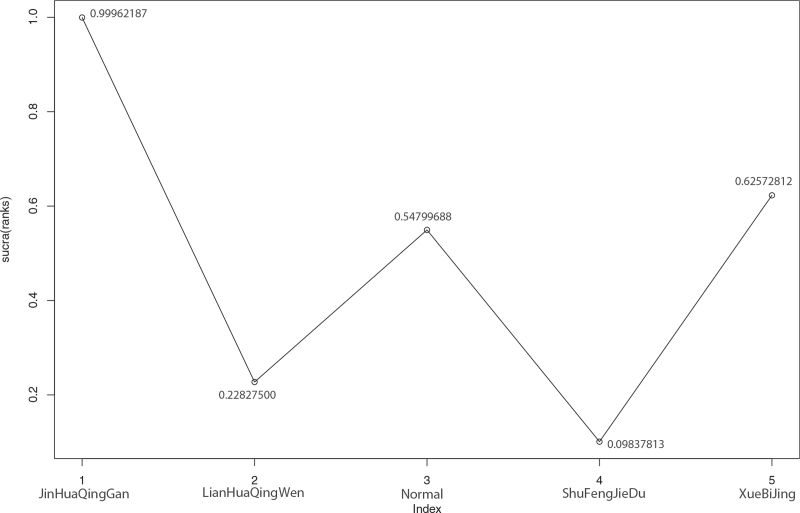
Sucra (rank) of adverse reactions of 4 kinds of traditional Chinese Medicine. 1. JinHuaQingGan (sucra [rank] = 0.99962187), 2. LianHuaQingWen (sucra [rank] = 0.22827500), 3. Normal (sucra [rank] = 0.54799688), 4. ShuFengJieDu (sucra [rank] = 0.09837813), 5. XueBiJing (sucra [rank] = 0.62572812).

Among the 15 included articles, 7 reported the incidence of adverse reactions of TCM in the treatment of COVID-19, including Jinhua Qinggan in 1 case,^[9]^ Lianhua Qingwen in 2 cases,^[15,17]^ Shufeng Jiedu in 3 cases,^[20–21,23]^ and Xuebijing in 1 case.^[22]^ According to the results of R software network meta-analysis, the incidence of adverse reactions of Lianhua Qingwen (OR = –0.49, 95%CI [–0.94,–0.05]) and Shufeng Jiedu (OR = –0.86, 95%CI [–1.89,0.09]) were lower than those of the conventional treatment group, and the difference was statistically significant. Jinhua Qinggan (OR = 39.93, 95%CI [5.09,126.3]) had a higher incidence of adverse reactions than the conventional treatment group and other TCM groups, and the difference was statistically significant. In terms of ranking order, Jinhua Qinggan > Xuebijing > Normal (conventional treatment) > Lianhua Qingwen > Shufeng Jiedu. Shufeng Jiedu and Lianhua Qingwen had the lowest incidence of adverse reactions, while Jinhua Qinggan had the highest incidence of adverse reactions. See Table 16 and Figure 16 for details.

**Table 16:** NMA of adverse reaction rate of 4 kinds of traditional Chinese medicine.

JinHuaQingGan	**–40.45 (–126.8, –5.58)**	**–40.83 (–127.1, –5.92)**	**–38.95 (–125.5, –3.85)**	**–39.93 (–126.3, –5.09)**
**40.45 (5.58, 126.8)**	LianHuaQingWen	**–0.37 (–1.49, 0.68)**	1.43 (–1.25, 5.01)	0.49 (0.05, 0.94)
**40.83 (5.92, 127.1)**	0.37 (–0.68, 1.49)	ShuFengJieDu	1.81 (–1.02, 5.51)	0.86 (–0.09, 1.89)
**38.95 (3.85, 125.5)**	–1.43 (–5.01, 1.25)	–1.81 (–5.51, 1.02)	XueBiJing	–0.93 (–4.5, 1.71)
**39.93 (5.09, 126.3)**	**–0.49 (–0.94, –0.05)**	**–0.86 (–1.89, 0.09)**	0.93 (–1.71, 4.5)	Normal

## 4 Discussion

A total of 15 studies were included in this study, including 7 RCTs and 8 retrospective studies. As the disease is caused by a novel coronavirus which was never detected before and it is highly contagious, but at the beginning, human beings did not have enough awareness about the disease, and it is still in the exploratory stage in the treatment plan now. And considering the ethical requirements, the prospective study is less than retrospective analysis. The Chinese medicines recommended by China's Novel Coronavirus Pneumonia Diagnosis and Treatment Plan (Trial Seventh Edition)^[6]^ were mostly used in mild, common, and clinical observation period COVID-19, so the primary outcomes were clinical efficiency (8 studies), and no mortality. The secondary outcomes were main common symptoms (fever, cough, and fatigue), improvement of lung imaging (CT), severe conversion rate, negative conversion rate of viral nucleic acid, and incidence of adverse reactions.

### 4.1 Effectiveness and safety analysis

The results of this study shows that Chinese medicine, as an adjuvant therapy combined with conventional treatment, is effective in the treatment of mild and common type of COVID-19 patients, which is manifested in improving the clinical efficiency, improving the main symptoms (fever, cough, fatigue disappearance rate and disappearance time), reducing severe conversion rate, and improving lung imaging. Therefore, the advantages of Chinese medicine as adjuvant therapy lie in the improvement of clinical efficacy and symptom group, and it can prevent patients from transforming to severe disease. After subgroup analysis of RCT and retrospective cohort study, the results show that there is no significant difference between them, which proves the reliability of the results. For the publication bias of clinical efficiency, the results of Begg test analysis showed that there was no bias, and the sensitivity analysis showed that the results were relatively stable, which furtherly proved that the results of this study were reliable. Toujie Quwen, Shufeng Jiedu, and Lianhua Qingwen showed obvious advantages in improving clinical efficiency, among which Toujie Quwen was the best. In terms of improving the main symptoms (fever, cough, fatigue disappearance rate and disappearance time), Shufeng Jiedu was the best. In terms of improving lung imaging, the CT improvement rate of Lianhua Qingke and Xuebijing was the highest. In terms of reducing the severe conversion rate, Lianhua Qingke had the lowest conversion rate. In terms of improving the negative conversion rate of nucleic acid of the virus, although the curative effect of TCM used as adjuvant therapy at the same time is not obvious compared with conventional treatment, the efficacy of a single Chinese medicine as adjuvant therapy is still higher than that of conventional treatment, and Reyanning had the best nucleic acid negative conversion rate. For the incidence of adverse reactions in this study, generally speaking, the safety of TCM as adjuvant therapy is not obvious compared with conventional treatment, but from the safety of single Chinese medicine, the incidence of adverse reactions of Shufeng Jiedu and Lianhua Qingwen is lower than that of conventional treatment while the incidence of adverse reactions of Xuebijing has no difference compared with conventional treatment. And the incidence of adverse reactions of Jinhua Qinggan is higher than that of conventional treatment.

Therefore, in terms of clinical efficacy and safety, Shufeng Jiedu and Lianhua Qingwen have obvious advantages. Although Lianhua Qingke has certain advantages in improving lung imaging and reducing severe conversion rate, its safety needs to be further determined. Reyanning has the best effect on the negative conversion rate of nucleic acid, but its therapeutic effect on other indicators of this study was less reported, so its efficacy and safety need to be further clarified.

### 4.2 Limitations of inclusion studies

According to the inclusion and exclusion criteria in the study, the 15 literatures included in this study all described the treatment group, control group, and outcome indicators in detail, but there are still some problems: 7 RCT studies reported random methods, but 4 studies only mentioned random number table method, and did not give a clear generation of random methods and 1 study only mentioned random methods. All RCT studies did not report the use of allocation concealment and blind method. Almost all RCT studies reported no loss of follow-up cases except Duan et al^[9]^ who reported 21 missing cases, but did not describe the specific treatment. Therefore, the literature reports are only with average quality, which suggests that there may be a certain degree of bias in the literature included in this study. No follow-up or long-term follow-up was found in 8 retrospective studies, and the reported results were not evaluated, so the quality of the literature was medium. In most studies, only the main indicators and some secondary indicators were counted. The evaluation indexes were not complete, and the evaluation criteria were not completely the same. Therefore, the results of this study may have some clinical heterogeneity. Most studies did not report the laboratory indicators of patients, such as nucleic acid negative rate, white blood cell count, lymphocyte%, C-reactive protein, etc, which will affect the overall quality of the included statistics. The epidemic situation still continues at present, the sample size of all the included studies is not large, most of them are single center studies, and there are certain clinical heterogeneity, such as course of treatment, course of disease, medication method, drug dosage form, etc due to medical ethics and other reasons, these will affect the reliability of the results. The literatures included in this study are all domestic studies, of which only 1 study of Hu et al^[15]^ is English literature, so it would produce large language bias which may affect the conclusion and extrapolation of meta-analysis. In the 15 studies, the TCM treatment of 5 studies was Lianhua Qingwen, while the involvement of other 7 kinds of TCMs was limited (1 or 2 of each). Thus the results of meta-analysis might be affected by this feature of the data imbalance.

## 5 Conclusion

As of January 21, 2021, the COVID-19 epidemic has caused more than 97.48 million infections and over 2.08 million deaths in more than 220 countries and regions around the world. The World Health Organization listed it as a public health emergency of international concern, and global politics and economy were greatly impacted by this epidemic. It has brought great damage to the physical and mental health of people around the world, and has brought immeasurable losses to globalization. Many countries will continue to be threatened and devastated by novel coronavirus pneumonia before the vaccine can be formally put into widespread clinical use and the effectiveness of the vaccine can be confirmed. It has far surpassed severe acute respiratory syndrome and Middle East respiratory syndrome in terms of the number of infections and the spatial scope of epidemic areas, and poses a serious threat to global public health.^[25]^ In this study, TCM as an adjuvant therapy combined with conventional treatment has better curative effect on mild and common type of COVID-19 patients. It has obvious effect in improving clinical efficiency, improving main symptoms (fever, cough, fatigue disappearance rate and its disappearance time), reducing severe conversion rate and improving lung imaging compared with conventional treatment. Therefore, the advantages of TCM as an adjuvant therapy lie in the improvement of clinical efficacy and symptom group, and it can prevent patients from transforming into severe cases. However, further analysis and research are needed to improve the safety and adverse reactions of patients. In terms of clinical efficacy and safety, Shufeng Jiedu and Lianhua Qingwen have obvious advantages, which are worthy of clinical promotion; although Lianhua Qingke has certain advantages in improving lung imaging and reducing severe conversion rate, its safety needs to be further determined. Reyanning has the best effect on the negative conversion rate of nucleic acid, but its therapeutic effect on other indicators of this study is less reported, so its efficacy and safety need to be further clarified. In addition, Jinhua Qinggan has a high incidence of adverse reactions, which should be used according to the actual situation. As the current global epidemic situation is still very serious, the successful experience of TCM used as an adjuvant therapy of conventional treatment in China is worth promoting to the world. In the existing clinical studies of TCM as intervention treatment of COVID-19, RCTs are relatively few, and retrospective studies account for a slightly larger proportion. It seems that there are a lot of studies, but there are not many studies that can be used for integrated analysis to furtherly play its research value, and therefore the level of evidence quality is generally. Of course, it is not easy to carry out clinical research in the current tense epidemic situation and arduous anti-epidemic work environment. However, in order to improve the evidence-based value of clinical research of TCM, it is suggested that local research groups should establish a multicenter controlled clinical trial on the existing basis, expand the efficacy comparison between conventional treatment and combined treatment of TCM and conventional treatment, so as to explore the clinical efficacy evidence of Chinese medicine intervention in COVID-19.

## Author contributions

This study is initiated by Xiaozheng Wu.

Xiaozheng Wu and Wen Li were involved in the design of the study and the interventions of the protocol.

Xiaozheng Wu will develop the search strategies, conduct data collection, and analyze independently.

Zhong Qin, Lei Xue, Gao Huang, and Zhenliang Luo will revise it.

All authors have approved the final manuscript.

**Conceptualization:** Xiaozheng Wu.

**Data curation:** Xiaozheng Wu.

**Formal analysis:** Xiaozheng Wu.

**Funding acquisition:** Xiaozheng Wu.

**Investigation:** Xiaozheng Wu.

**Methodology:** Xiaozheng Wu, Wen Li.

**Project administration:** Xiaozheng Wu, Lei Xue.

**Resources:** Xiaozheng Wu.

**Software:** Xiaozheng Wu, Wen Li.

**Supervision:** Yunzhi Chen.

**Validation:** Zhenliang Luo.

**Visualization:** Gao Huang.

**Writing – original draft:** Xiaozheng Wu.

**Writing – review & editing:** Xiaozheng Wu, Zhong Qin.
